# Aetiology, treatment patterns and long-term outcomes of tooth avulsion in children and adolescents

**DOI:** 10.12669/pjms.292.3283

**Published:** 2013-04

**Authors:** Huseyin Karayilmaz, Zuhal Kirzioglu, Ozge Erken Gungor

**Affiliations:** 1 Dr. Huseyin Karayilmaz, Assistant Professor, Department of Pedodontics. Akdeniz University, Faculty of Dentistry, Antalya, Turkey.; 2 Dr. Zuhal Kirzioglu, Professor, Department of Pedodontics. Suleyman Demirel University, Faculty of Dentistry, Antalya, Turkey. Department of Pedodontics. Akdeniz University, Faculty of Dentistry, Antalya, Turkey.; 3 Dr. Ozge Erken Gungor, Assistant Professor, Department of Pedodontics. Akdeniz University, Faculty of Dentistry, Antalya, Turkey.

**Keywords:** Traumatic Dental Injuries, Tooth Avulsion, Replantation

## Abstract

***Objective:*** Tooth avulsion constituting an emergency for children and adolescents necessitates management approaches ensuring the survival of avulsed teeth. The aim of this study was to determine the causes of tooth avulsion and to examine some factors affecting the clinical & radiographic assessment of their prognosis after replantation.

***Methodology:*** The study sample was created by using archival records of patients who were referred to the Suleyman Demirel University, Faculty of Dentistry, Department of Pedodontics, with complaint of traumatic injuries, between December 1999 and 2008. The information about age, gender, time and cause of the injury, number of affected teeth, the root maturation level (mature/immature), vitality of the affected teeth, condition of supporting tissues, extra-oral time of avulsed teeth, storage media, time of replantation, type and duration of splinting, and healing process was obtained from the patients’ records.

***Results:*** The sample consisted of 66 traumatized children who had a total of 93 avulsed anterior permanent teeth. The age of these patients ranged from 6 to 16 years and the 9- and 10-year-old group had the highest incidence (n=25). The most frequent causes were falls (n=24; 36.4%). Thirty-three out of a total of 93 avulsed teeth (35.5%) were replanted. Of the 33 replanted teeth, 3 (9.1%) were stored in milk and 25 were stored in dry media (n=25; 75.8%). Fifteen teeth (45.5%) were replanted within 30 minutes after the injury. After clinical and radiographic evaluation a total of 12 replanted teeth (36.4%) were considered as failed. Ten of the replanted teeth had to be extracted due to progressive root resorption. Statistical analysis showed no significant relationship between the successes of replanted teeth with extra-oral period, storage media, root formation stage, and additional traumas to the supporting tissues (p>0.05).

***Conclusion:*** In this study, during the 9-year period, it was determined that 5.87% of all traumatic dental consisted of avulsion injuries. Thirty-three avulsed teeth in 26 patients were replanted, and 12 replanted teeth were revealed as failures. A high rate of success can be obtained when the avulsed teeth are kept under wet conditions and brought to a dental clinic as soon as possible.

## INTRODUCTION

Traumatic dental injuries represent one of the most common reasons for emergency appointments, and ensuring the survival of traumatized teeth is one of the main responsibilities of dentists and also physicians. However, in severe cases like avulsion injuries, survival of the tooth is not always possible. “Avulsion” is used to describe a situation in which a tooth has been removed from its socket as a result of severe trauma. The treatment of avulsed teeth is very complicated because the periodontal ligament (PDL) fibers, the neurovascular bundle at the root apex, the cement layer of the tooth, alveolar bone, and the gingiva are all damaged. In these circumstances, the prognosis of avulsion cases is very poor and many factors have been thought to affect their success rate. Because of the injury’s complicated nature, there are few published studies about the management and prognosis of avulsed teeth.^1^^-^^6^

The aim of this study was to determine the causes of tooth avulsion and to examine some factors affecting the clinical & radiographic assessment of their prognosis after replantation.

## METHODOLOGY

The study sample was created using archival records of patients who were referred to the Suleyman Demirel University, Faculty of Dentistry, Department of Pedodontics, with complaint of traumatic injuries from the cities of the West-Mediterranean region of Turkey between December 1999 and December 2008. The study had been approved by the ethics council of the Akdeniz University, Faculty of Medicine and informed consent of the patients or parents had been taken. The dental treatments of 1124 patients who had experienced various types of traumatic dental injuries were performed in our clinics during a 9-year period. The sample consisted of 66 traumatized children who had a total of 93 avulsed anterior permanent teeth. The information about age, gender, time and cause of the injury, number of affected teeth, the root maturation level (mature/immature), vitality of the affected teeth, condition of supporting tissues, extra-oral time of avulsed teeth, storage media, time of replantation, type and duration of splinting, and healing process was obtained from the patients’ records. Patients were classified in three groups according to their age at the time of the trauma (Table-I). 

**Table-I:** The distribution of patient’s ages and age groups according to gender

*Age* *Groups*	*Ages* *(Years-old)*	*Girls*	*Boys*	*Total*
Group I	6-9	15	13	28
Group II	10-13	08	22	30
Group III	14-16	03	5	08
Total		26	40	66

**Table-II:** The healing status of replanted teeth was evaluated in 4 different groups.^7^

		*Clinically*	*Radiographically*
Group A	Healing with a normal periodontal ligament; is characterized by complete regeneration of the periodontal ligament.	The tooth is in a normal position Normal mobility Normal percussion tone Normal periodontal ligament space No signs of root resorption	
Group B	Healing with surface resorption (repair-related resorption); is characterized by localized areas along the root surface which show superficial resorption lacunae repaired by new cementum	The tooth is in a normal position Normal mobility Normal percussion tone	Usually not disclosed radiographically Small excavations on root surface Normal periodontal ligament space width
Group C	Healing with ankylosis (replacement resorption); represents a fusion of the alveolar bone and the root surface.	The tooth is immobile The percussion tone is high, differing clearly from adjacent non-injured teeth	Disappearance of the normal periodontal space Continuous replacement of root substance with bone
Group D	Healing with inflammatory resorption (infection-related resorption); is characterized by bowl-shaped resorption cavities in cementum and dentine associated with inflammatory changes in the adjacent periodontal tissue	The tooth is sensitive to percussion Percussion tone is dull The tooth is extruded and loose Radiolucent bowl shaped cavitations along the root surface Corresponding excavations in the adjacent bone	

**Table-III:** The healing status of 33 replanted teeth according to extra-oral time and storage media

*Healing* *Status*	*Extra-oral Time*	*Storage Media* *(n=33 teeth)*				
		*Milk*	*Dry*	*Saliva*	*Others*	*Total*
Successful (Group A)	30 min	0	2	2	0	4
	30 min-2h	1	0	0	0	1
	2h ↑	1	1	0	0	2
	Total	2	3	2	0	7
Acceptable Success (Group B)	30 min	0	1	1	1	3
	30 min-2h	0	2	0	1	3
	2h ↑	1	3	0	0	4
	Total	1	6	1	2	10
Un-successful (Group C and D)	30 min	0	6	0	0	6
	30 min-2h	0	3	0	0	3
	2h ↑	0	3	0	0	3
	Total	0	12	0	0	12
Not Followed	30 min	0	2	0	0	2
	30 min-2h	0	2	0	0	2
	Total	0	4	0	0	4

The 26 patients who were treated with replantation of 33 teeth during the period of 1999–2008 were recalled for follow-up evaluations. During clinical examination of the replanted teeth, pulp vitality, tooth mobility changes, tooth discoloration and infra-position, percussion sounds, pain on palpation, and presence of fistulae were examined. The indicated dental radiographs were obtained from each patient.

The healing status of the replanted teeth was evaluated in 4 different groups according to the healing modalities in the PDL space as expressed by Andreasen et al.^7^ (Table-II). The replanted teeth, which were designated as Groups C and D, were revealed as failure, while Group A was revealed as a successful in this study.

The collected data were analyzed using SPSS for Windows (Version 17.00; SPSS Inc., Chicago, IL, USA). Cross-tabulations with Fisher’s Exact Test were performed to examine the relationship between the clinical and radiographical findings and the treatment results. Significance was noted at the 0.05 level.

## RESULTS

During the 9-year period, the patients referred to our clinic due to avulsion injury were 5.87% (66/1124 children with 93 avulsed permanent teeth) of all traumatic dental injury patients. The age of these patients ranged from 6 to 16 years, with an average age of 10.18 ± 2.5 years. The 10-(n=14), 9-(n=11), and 11-(n=8) year-old groups had the highest incidence of avulsion injuries, respectively (Table-I). It was observed that male patients (n=40) experienced more avulsion injuries than female patients (n=26) (boys-to-girls ratio, 1.5:1).

The causes of avulsion injuries were falls (n=24; 36.4%), traffic accidents (n=15; 22.7%), bicycle accidents (n=12; 18.2%), collisions (n=6; 9.1%), and other causes (n=9; 13.6%) respectively. The difference between causes and gender (p=0.722), causes and age groups (p=0.164) was not statistically significant. The average number of avulsed teeth per child was 1.4, and 69.7% of the children were found to have one avulsed tooth, while 19.7% presented with two and 10.6% presented three avulsed teeth. All affected teeth were the anterior permanent teeth except for a lower left first premolar. The majority of avulsed teeth were maxillary centrals (left=37, 39.8%, right=35, 37.6%), followed by the maxillary lateral incisors (left=8, 8.6%, right=3, 3.2%). No statistical difference was found between cause and number of avulsed teeth (p=0.663).

Thirty-three avulsed anterior teeth were replanted in 26 patients. In 40 patients, 60 avulsed teeth were treated without replantation, because the avulsed teeth were not brought in along with the patient. These patients were treated with space maintainers until they reached the appropriate age for orthodontic and prosthetic (fixed bridge, implant etc.) rehabilitation.

Of the 33 replanted teeth, three (9.1%) were stored in milk and three (9.1%) were stored in the patients’ oral cavity. But, all the other replanted teeth except for two (stored in ice and alcohol) were stored in dry media like a paper napkin (n=25; 75.8%).

Although 15 teeth were replanted within 30 minutes and 9 teeth were replanted in 30 min-2 hour after the injury, 9 teeth were treated with late replantation (2–10 h). All the teeth were replanted in our departmental clinics except for the two teeth that were treated with late replantation in a dentistry clinic at the place of the accident. The healing statuses were 4/15 teeth that were replanted within 30 minutes in Group A, and 3/15 teeth in Group B. But, replantation of 6/15 teeth failed (Group C and D) and 2/15 teeth could not be followed up (Table-III).

It was found that 39.4% (n=13) of the replanted teeth had incomplete root formation although 60.6% (n=20) of the teeth had complete root formation. In 16 replanted teeth (48.5%), additional injuries in the supporting and neighboring tissues were identified. Semi-rigid splints were used to immobilize all the replanted teeth without any additional injuries in supporting tissues for 1–2 weeks. The endodontic treatment with calcium hydroxide of 28/33 teeth (84.8%) was initiated intra-orally. But, the endodontic treatment of four teeth could not be finished because the treatment was done by another dentist. The endodontic treatment of 5/33 teeth (15.2%) was performed extra-orally, due to severe additional injuries in the supporting tissues.

The four replanted teeth in four patients could not be followed. The observation period for 22 children (29 teeth) ranged from 1 to 8 years (mean=32.73±27.01). Although, 6 children (6 teeth) could be followed for more than 5 years, 8 children (9 teeth) could be followed for 2-5 years and 8 patients (14 teeth) could be followed for less than two years.

A total of 12/33 replanted teeth (36.4%) were revealed as failures. Of these, 4/12 teeth failed due to ankylosis (Group C) and 8/12 teeth due to the inflammation resorption (Group D). Among the other cases, 7/33 teeth (21.2%) revealed in Group A and 10/33 teeth (30.3%) revealed in Group B (Table-III). Ten of the replanted teeth had to be extracted due to progressive root resorption. Apical maturation levels at the time of replantation of half of the extracted teeth were immature. Infra-occlusion was diagnosed in only two teeth.

Statistical analysis showed no significant relationship between the successes of replanted teeth with extra-oral period (p=0.771), storage media (p=0.058), root formation stage (p=0.983), and additional traumas to the supporting tissues (p=0.314). During the follow-up of the replanted teeth, 10 of 33 replanted teeth had been exposed to new dental traumas. There was a statistically significant relationship between repeated traumas and the success rate of replanted teeth (p=0.02).

## DISCUSSION

The avulsion of teeth following traumatic injury is relatively infrequent, ranging from 0.5 to 6.2% of all traumatic injuries in permanent dentition.^7^^,^^8^ During the 9-year period, 5.87% of all traumatic dental injuries referred to our clinic consisted of avulsion injuries.

Management of avulsion of the permanent dentition often presents a challenge. The prognosis depends on the measures taken at or immediately after the time of the avulsion. Replantation is the only treatment choice, but careful assessment of the cases is of up most importance for the avulsed tooth to be successfully replanted.^9^ All the avulsion cases (26 children/33 teeth) were evaluated carefully and replantation was performed for all avulsed teeth brought to our clinics. Similar with our study, Petrovic et al^10^ replanted all teeth brought in with the patient. In our study, 64.5% of avulsed teeth (60/93 teeth) were lost at injury, due to panic and ignorance of the children or parents/caregivers. This percentage was reported at 70% (63/90 teeth) by Tzigkounakis et al^2^, as 56.25% (18/32 teeth) by Kinoshita et al^11^, and as 48.38% (30/62 teeth) by Petrovic et al.^10^

The treatment outcome of replanted teeth can be influenced by several factors such as extra-alveolar period, storage medium, stage of root formation, and concomitant dentoalveolar injuries.^2^^,^^4^^,^^7^^,^^10^

Before replantation, of an avulsed permanent tooth in particular, the extra-alveolar period and storage medium should be considered. The time until replantation ranged between 30 minutes and 10 hour, in our study. Kinoshita et al^11^ and Tzigkounakis et al^2^, reported this time interval as 30 minutes to 3/3.5 hour, while Petrovic et al^10^ reported this as 15 minutes to 9 hour, in accordance with our study. The extra-alveolar period should ideally be a maximum of 20 to 30 minutes for the best prognosis.^7^^,^^9^ However, immediate replantation of an avulsed tooth after injury is not always possible, and alternative treatments should be employed in the search for satisfactory outcomes for patients, such as the accomplishment of late replantation.^12^ The procedure of late replantation is contraindicated by some authors.^13^ But, according to Andreasen et al^14^, even teeth kept dry for a long period should be replanted. A case that was successfully replanted two days after the trauma was reported recently.^12^ In our study, 9 teeth in 6 patients were replanted within 2-10 hour after the injury, according to the recommendations of Andreasen et al^14^, and the International Association for Dental Traumatology guidelines.^9^ The healing statuses of these teeth were as follows: 2 teeth were revealed in Group A and 4 teeth revealed in Group B. But, the replantation of 3 teeth failed (Group C and D) (Table-III).

Storage medium was another crucial factor for successful replantation, and many storage media have been recommended (Hank’s Balanced Salt Solution [HBSS], ViaSpan, Eagle’s Medium, milk, sterile saline, etc.).^15^^-^^18^ ViaSpan and Eagle’s Medium provide good storage environments, but both are very expensive, are not packed for individual use, and must be refrigerated. HBSS was found to be the most suitable solution among the recommended storage solutions.^7^^,^^18^ HBSS stores and preserves avulsed teeth for at least 24 hour and needs no refrigeration. Despite this fact, this media is not commercially available in markets, drug stores, and pharmacies in our country. Consequently, parents or caretakers of the children must choose an alternative media like milk, saline, or water, to transport an avulsed tooth. Milk is an easy and inexpensive method for storage of an avulsed tooth and has a compatible osmolality with the PDL cells. However, it does not contain the necessary nutrients to maintain the PDL cells and is not effective for storing the avulsed tooth for more than 2–3 hour. Additionally, milk needs to be kept refrigerated during transport for the best prognosis.^15^^-^^18^ In our study, it was found that only three teeth (9.1%) were stored in milk and two of these teeth showed healing with normal PDL and the other tooth showed healing with surface resorption regardless of extra-alveolar time. But, it was determined that the most of the replanted teeth were stored in dry media (n=25; 75.8%). Half of the failed cases (n=6) that were replanted within 30 minutes after the injury were stored in dry media.

An increased incidence was determined between the ages of 9 and 10 years, which is in agreement with other studies.^2^^,^^4^ Our finding that falls were the most frequent cause of trauma in all ages is also generally supported by other studies.^2^^,^^4^^,^^8^

Maxillary central and lateral incisors (n=80; 86%) were the teeth most affected by avulsion injuries because of their protrusive and vulnerable positions. This finding corroborates the earlier findings of researchers.^2^^,^^4^^,^^7^^,^^19^^,^^20^ The majority of patients presented with only one avulsed tooth (n=69; 7%), and the number of avulsed teeth per patient was 1.4 in our study. This rate ranged between 1.5 and 2.0.^2^^,^^4^^,^^20^

## CONCLUSION

In this study, during the 9-year period, 5.87% of all traumatic dental injuries referred to our clinic consisted of avulsion injuries. Thirty-three avulsed teeth in 26 patients were replanted, and 12 replanted teeth were revealed as failures. A high rate of success can be obtained when the avulsed teeth are kept under wet conditions and brought to a dental clinic as soon as possible. The importance of quick replantation or the need for storage media after tooth avulsion appears to be often less recognized by not only parents or schoolteachers but also by general dentists or physicians. It is necessary to educate the public regarding the possibility of replanting the avulsed permanent tooth of a child and that the preferable preservative media are fresh milk or HBSS (if available).
